# Indigenous peoples’ territorial sovereign in the Amazon must be respected

**DOI:** 10.1016/j.lana.2022.100331

**Published:** 2022-07-14

**Authors:** The Lancet Regional Health– Americas

“This here is my life, my soul. If you take the land away from me, you take my life.” - Marcos Veron, Guarani-Kaiowá tribe leader, murdered in 2003.

Indigenous people have been fighting for their lands since the Europeans arrived in Brazil more than 500 years ago. Their relationship with the land is not about power or possession. Their territory is tied to their identity, culture, and spiritual beings. It is who they are. After decades of genocides that decimated hundreds of Indigenous communities, the 1988 Brazilian Constitution recognised Indigenous people as the first and natural owners of the land, defined that any explorative incursion must be authorised by the National Congress after listening to the communities involved, and prohibited eviction of Indigenous people from their lands. However, those lands are only protected after being recognised, a process called “demarcation”. In his first week in office, Brazilian President Bolsonaro promised his voters that, during his government “there would not be one more centimetre of land demarcated for indigenous peoples”. For Indigenous leaders, this was the moment he declared war against them.

The war against Indigenous people is not only another racist rampage, but also a political movement. 98.5% of the 690 recognised Indigenous territories lie in the Amazon and account for about 13% of Brazil's land mass. What is at stake here is the right to explore the natural resources and use the land for agricultural business, mining, and oil drilling. Deforestation coupled with hydroelectric dams, cattle ranching, mines, and roads expose Indigenous communities to forced contact, armed conflicts, and diseases to which they have no immunity, such as influenza, measles, and tuberculosis. Indigenous people have a deep, spiritual, and cultural connection with their land, and forced displacement often leads to a heavy psychological burden. In the Guarani people, who have lost almost their entire territory to cattle ranches and sugar cane plantations, the suicide rate is over 19 times the national average. By refusing to recognise Indigenous territories, the Government forces their exposure to external people, facilitates land appropriation, and incites displacement from their traditional territories against their will, threatening their physical and mental health.

The UN Declaration on the Rights of Indigenous Peoples (UNDRIP) states that “Indigenous peoples have the right to the lands, territories and resources which they have traditionally owned, occupied or otherwise used or acquired” (Article 3); a right ratified by the Inter American Court of Human Rights and reinforced by the International Labour Organization's Indigenous and Tribal Peoples Convention 169. In 2007, when the declaration was voted, Brazil was one of the greatest supporters of UNDRIP, considered a long-awaited achievement to support and protect Indigenous people's rights. However, in recent years, lobbying from farmers and international mining companies has pushed for law amendments and decrees to restrict demarcation and allow the exploitation of natural resources on traditional Indigenous lands.

The movement to legalise deforestation and land appropriation also encourages illegal activities. According to MapBiomas calculations, illegal mining in the Yanomami Indigenous Land (one of the biggest demarcated Indigenous territories in the Amazon) grew by 3350% from 2016 to 2020. Illegal mining frequently uses mercury, which pollutes the rivers and contaminates the fish. A study with Munduruku Indigenous adults in the Amazon basin alerted to the neurological impacts of chronic intoxication with mercury in those communities. Invasion of Indigenous lands infringe additional risks to women and girls, who frequently report being sexually assaulted and exploited by miners and lodgers. Furthermore, the latest report from the Amazonian Environmental Research Institute (IPAM) shows that deforestation in the Amazonian biome between August, 2018, and July, 2021, was 56.6% higher than the corresponding period in 2015–18.

Indigenous rights advocates, environmentalists, and journalists have been denouncing the rise in land invasion, illegal exploitation, and their environmental consequences, and many have been silenced. In fact, the rise in illegal activities in the Amazon was accompanied by a rise in violence and armed conflicts. The recent assassination of the Indigenous rights advocate Bruno Pereira and British journalist Dom Phillips while documenting illegal activities in the Brazilian Amazon shocked the world but, sadly, that was not an isolated case. In 2020 alone, 202 human rights defenders were murdered in countries in the Amazon basin (Bolivia, Brazil, Colombia, and Peru), with 69% of them being leaders working to defend the environment and the rights and territories of Indigenous people.

There are about 305 tribes living in Brazil today, accounting for almost 900,000 people (0.4% of Brazil's population). The Brazilian Amazon is also home to over 100 groups of uncontacted people—the largest number in the world, and the Government's plan to destroy and commodify Indigenous territories is an open threat to their right to exist. It is not only imperative that the Government halts any further movement to restrict Indigenous rights, but also crucial to actively commit to improving and protecting their rights to self-determination (the right to determine one's own social, cultural, and economic development) and territorial sovereignty, recognising that their lives must always prevail against any economic or political gain.

